# Disconnected Lives: Social Networks and Emotional Regulation in Domestic Dogs

**DOI:** 10.3390/ani16030398

**Published:** 2026-01-27

**Authors:** Agnieszka Grynkiewicz, Anna Reinholz, Kamil Imbir

**Affiliations:** Department of Psychology, University of Warsaw, 02-097 Warsaw, Poland; areinholz@psych.uw.edu.pl (A.R.); kimbir@psych.uw.edu.pl (K.I.)

**Keywords:** urban dogs, social fragmentation, emotional dysregulation, social co-regulation, canine welfare

## Abstract

Dogs are deeply social, built to stay in touch with others of their kind. In cities, though, most now live as single dogs. Housing rules, work schedules, and constant supervision have constrained their social environment. They still meet other dogs, but the meetings are short, managed, and rarely turn into real bonds. This review tries to pull together what is known about how such limited contact affects canine welfare and emotional balance. The sources come mostly from ethology, psychology, and urban studies, published between 2010 and 2025, and include comparisons between urban pets and free-ranging dogs that still organise their own social lives. Across studies, the pattern is similar: when dogs lose steady companions, they also lose the kind of social buffering that once helped them recover from stress. Over time, this does not always look like distress—more often it shows up as quiet tension, watchfulness, or an overdependence on human cues. The evidence points to social deprivation as a slow, structural welfare issue rather than an occasional problem. Meaningful improvement may therefore require moving beyond control and training alone, toward conditions that allow dogs to form small, stable circles of familiar peers that support lower arousal and more reliable recovery.

## Simple Summary

Domestic dogs are highly social, yet most pet dogs live in single-dog households and experience limited opportunities for sustained intra-specific bonds. They rarely have a chance to form lasting bonds with their own kind. Most studies still look at dogs in multi-dog homes or daycare, which is a small and unusual part of the population. It tells little about ordinary city dogs. Urban life adds limits of its own—leashes, lifts, small flats, crowded parks—plenty of meetings, but short and shallow ones. Evidence suggests that positive dog-to-dog contact can buffer stress, while poor early socialisation increases fear and reactivity. Comparisons with free-ranging dogs, which maintain flexible social networks, highlight the potential costs of the highly restricted social life of pet dogs. Free-ranging dogs, who still live in small flexible groups, show what is lost when this social system disappears. This review gathers available findings and points to an overlooked welfare issue: the quiet social deprivation of urban dogs, and what it means for both canine well-being and human–dog communities.

## 1. Introduction

Dogs were shaped by evolution among others of their kind, learning safety and balance through company [1,2]. Today most live surrounded by people, but often without cohabiting with another dog. The routines that shape human life—small homes, long absences, dense cities—have indirectly redrawn what social life means for them [3,4,5]. Once surrounded by other dogs and shared daily routines, many dogs now experience only brief or incidental contact with conspecifics. This integration into human families alongside restricted conspecific social life raises welfare concerns regarding the evolution of the human–dog bond. Today, more dogs live as urban companions than at any time before, and the effects of limited social contact can be seen in their everyday lives [6,7].

Over the past two decades, research on dog behaviour and cognition has grown quickly. Most of this work has focused on dogs’ attachment to humans and their emotional sensitivity to our signals [8,9,10]. For comparison, studies of free-ranging dogs describe social systems held together by tolerance—loose, shifting alliances that sometimes give way to avoidance or even conflict [11,12]. Still, such findings capture only one side of canine social life, we still do not know much about how pet dogs interact with other dogs in everyday settings. Most theoretical models still centre on the human–dog dyad, leaving the broader social setting—the one that shapes behaviour and emotion—largely unexamined [13,14]. Because of this narrow focus, we know far less about how limited peer contact affects the everyday welfare of urban dogs [10,13]. This review takes up that gap by combining psychological and ethological research with insights from urban studies. It looks at how everyday human routines and city design shape what dogs can do socially—who they meet, how often, and under what conditions [5,15]. We suggest that social ties act as emotional regulators; when those ties fragment, stability begins to falter [7,14,16]. The aim, then, is to shift focus: not to the dog as a solitary human companion, but to the dog as a social animal living within human-made worlds that keep changing around it [17].

## 2. Materials and Methods

The literature search was built around studies in canine ethology and social cognition, with supporting material drawn from demography and welfare research. The review focused on how social fragmentation and isolation shape the emotional stability of urban companion dogs.

### 2.1. Search Strategy and Scope

The main literature searches were carried out in October 2025. The review focused on peer-reviewed publications primarily from 2010 to 2025, a period marked by rapid growth in research on dog cognition, welfare, and human–animal relationships. Earlier foundational works—for example, those framing attachment, biopsychosocial, and polyvagal models—were included when they provided essential theoretical grounding.

Sources were retrieved from multiple databases and tools to ensure broad coverage: Web of Science, Scopus, PubMed, Google Scholar, and, where appropriate, Consensus and Research Rabbit to locate related or recently released works not yet indexed in standard repositories. Search terms were defined to capture key themes: emotional and social regulation, environmental and demographic influences, and the welfare outcomes linked to them. Keywords included “social co-regulation,” “emotional regulation,” “attachment theory,” “social buffering,” “single-dog households,” “urbanization,” “social fragmentation,” “free-ranging dog social structure,” “behavioral flexibility,” “social network analysis,” “relationship quality,” “methodological bias,” “micro-communities,” and “canine welfare.” Each search term returned between 50 and 300 results depending on specificity, with the highest yields for demographic and welfare-related keywords.

This review follows a narrative synthesis approach enriched with structured database searching; it is not a systematic review and does not aim to provide exhaustive coverage.

### 2.2. Screening and Inclusion Criteria

Preliminary searches returned around 250 publications. Following title and abstract screening for relevance to canine sociality and welfare, roughly 100 were selected for full-text review and inclusion in the synthesis. Eligible works were peer-reviewed and showed clear empirical or theoretical relevance to social regulation and fragmentation. Studies were excluded when they were anecdotal, purely descriptive, or focused narrowly on training without a defined methodological or theoretical framework.

To place the topic in a wider conceptual context, the corpus also included a small number of comparative studies on other social mammals (e.g., primates, rodents, elephants) and several human-based papers that served as conceptual anchors for understanding co-regulation and network dynamics. These comparative references were used to contextualise mechanisms rather than to expand the empirical scope.

### 2.3. Quantitative Overview and Limitations

The final manuscript included over 100 references, combining empirical findings with theoretical work. Most came from demographic and ownership studies, which provided the contextual foundation for the analysis.

This synthesis also inherits the constraints of the existing literature. Research on companion dogs remains largely Western in focus, centred on the human–dog relationship or specific behavioural scenarios [3,10,15]. As a result, everyday social life among dogs—the spontaneous interactions that occur outside structured or owner-mediated settings—is rarely documented [5,13,18]. Addressing this gap calls for a broader, multi-context approach to the ethology of urban dogs and future welfare research. The scope of available data, in turn, shaped how this review was structured and interpreted.

## 3. Theoretical Perspectives on Canine Regulation and Sociality

Domestic dogs are not only highly social but also regulatory animals. Their emotional balance depends on the stability of their social bonds and on the mechanisms that sustain those bonds over time. Understanding canine sociality requires more than counting interactions; it means asking how relationships regulate emotion and behaviour. This perspective sees dogs as individuals whose functioning is shaped by continuous processes of emotional and physiological co-regulation—systems in which both partners influence each other’s state in real time.

### 3.1. The Dog as a Social Regulator

Dogs have always lived with others. For most of their history, survival and reduced vigilance depended on company [1,19]. Groups offered protection, but also something less visible: emotional steadiness [1,2,8,10]. Social contact makes life predictable enough for the body to relax [2,8,20,21]. When that structure disappears, stress lingers and what should be short recovery becomes a long strain [6,22,23]. Being social is not a luxury—it is a physiological need that shapes how dogs recover and stay resilient [9,24,25,26,27]. In this context, stress is the organism’s response when environmental demands disturb homeostasis, often involving activation of the hypothalamic–pituitary–adrenal (HPA) axis. Social co-regulation is what happens when two (or more) partners continuously tune to each other’s arousal and behaviour, which supports recovery and emotional stability. Social roles are treated here as stable, functional behaviour patterns within a group (e.g., individuals that more often initiate care, interrupt escalation, or stabilise proximity), helping coordination and lowering tension.

In mammals, co-regulation comes from a mix of everyday social behaviours: repairing tension, being soothed by a familiar partner, sharing emotional states, and using touch to settle. Each does a small part, and together they help bring arousal down and keep relationships steady. These processes are supported by oxytocin-mediated modulation of the HPA axis and by neural circuits linking limbic and prefrontal regions, which integrate social cues with internal states [28,29,30]. Field studies across primates, elephants, equines, and rodents show that co-regulation is an evolutionarily conserved strategy: affiliative contact repairs tension, proximity buffers stress, and coordinated behaviour promotes group cohesion [31,32,33,34]. These patterns highlight that emotional stability in social species emerges from interaction rather than from individual control, and that early social experience calibrates the development of these mechanisms [35,36].

Dogs fit naturally into this framework. Their physiological states align with those of their guardians, including long-term cortisol synchrony [37,38], and proximity to a familiar human reliably reduces arousal even in dogs with adverse early histories [39,40]. Dogs also show dyadic regulatory processes with conspecifics: symmetry and turn-taking in play, rapid repair after minor conflicts, and flexible shifts in approach or withdrawal that reflect attention to the partner’s arousal. Social buffering occurs not only with familiar humans but also, in some contexts, with unfamiliar people, suggesting that regulatory competence extends beyond specific attachment bonds [41]. Together, the findings frame dogs as part of relational systems in which emotional stability comes from shared, coordinated behaviour—not from isolated responses.

In this paper, co-regulation is a lens for looking at how dogs hold their emotional balance in the broken-up social spaces of cities. This co-regulation framework moves the focus away from isolated acts toward the patterns that join them: steady partners, the tone of contact, the pace of recovery. This framing also shows how attachment and social networks relate to one another in the sections that follow.

### 3.2. Attachment and Co-Regulation Across Species

Attachment theory has profoundly shaped research on the dog–human bond. Adaptations of Ainsworth’s Strange Situation Test show that dogs maintain proximity to their caregivers and treat them as a secure base—attachment signs most visible during separation [2,8]. Similar attachment-like behaviours appear in intensively socialised wolves [42,43], confirming that emotional safety can cross species boundaries. Yet framing attachment through a child–parent lens leaves little room for reciprocal or peer-based forms of connection—relationships that in adult dogs may be more typical and often more adaptive.

Security does not have to mean dependence; stability can arise from autonomy and mutual trust [2,20]. Bonds between dogs are sometimes viewed as “weaker” simply because they provoke less visible distress during separation—though such calmness may reflect secure regulation rather than detachment. Most experimental designs still cast humans as the regulating partner and rarely examine how one dog steadies another—through proximity, touch, or small cues of reassurance. This asymmetry sustains an anthropocentric picture of attachment, even though studies of free-ranging groups and multi-dog households show clear patterns of social buffering among dogs themselves [44]. Physiological data add a parallel view. Cortisol and oxytocin levels in dogs and humans shift in synchrony during friendly contact [9,45], revealing a biobehavioural coupling that regulates arousal. The same principle operates in affiliative stability among dogs, linking social context with stress modulation—the essence of the biopsychosocial model [46]. In dogs, social context shapes stress responses and learning, while the steadiness of relationships supports behavioural health.

### 3.3. Social Stress Models

According to Polyvagal Theory [16], the sense of safety engages parasympathetic pathways that promote calm and inhibit defence. For dogs, the presence of trusted partners—human or canine—can buffer stress even in difficult contexts like shelters [20,47]. When predictability disappears or isolation lasts too long, these systems begin to wear down, keeping the animal in chronic vigilance [48]. Everyday life and environment shape these mechanisms further. Dogs that live with consistent partners and varied social contact usually show lower fear and anxiety [6,7]. Stable attachment supports welfare and social competence [8,10]. What counts is not dependency itself but the steadiness of relationships—the reciprocity that lets the nervous system move naturally between connection and rest.

Together, these findings define social safety as a biological need and set the stage for the next sections, which explore how such regulation functions—and where it breaks down—in the managed social worlds of domestic dogs.

### 3.4. Summary

Across these theoretical perspectives, one conclusion stands out: social relationships operate as regulatory systems. For dogs, attachment and social contact go beyond mere affection. They are mechanisms that modulate stress and integrate emotion into daily functioning. Theories of attachment, biopsychosocial interaction, and social safety all point in the same direction—dogs need steady, meaningful partners to stay emotionally stable.

## 4. Global and Demographic Context of Dog Social Life

### 4.1. Global Dog Populations and Ownership Patterns

Estimates of the global dog population vary widely, but most of the world’s dogs are not confined pets. Demographic analyses place the total number between 700 million and more than 1 billion individuals, of which roughly 75–80% live as free-ranging, community, or loosely owned dogs [49,50,51]. This group includes both unowned street dogs and semi-owned individuals that maintain contact with people while living largely independently.

Following Boitani’s classification of canine populations [52], dogs can be situated along a spectrum of human dependence, from feral and semi-independent village dogs to fully restricted companion dogs. This review focuses on the social consequences of shifting from free-ranging social organisation to highly managed urban pet life (Table 1).

In Western countries, dogs have become part of everyday family life: between 20 and 50 percent of households include at least one dog [58,59,60]. This scale shows that canine sociality is woven into urban culture itself. Elsewhere, the picture differs. In many low- and middle-income countries, dogs still live largely free. Surveys from India and Brazil record dense street populations that blend into both city neighbourhoods and rural communities—animals maintaining territories and small social groups within human environments [53,61]. Across much of Asia and Africa, the trend repeats: free-living dogs frequently make up over 80 percent of local populations [51]. These dogs move within networks of tolerance and cooperation, interacting regularly with people and with one another. Wealthier countries present the opposite picture. Over recent decades, dogs have shifted almost entirely into conditions of confinement and individualised ownership. In the United States, the United Kingdom, and most of Europe, nearly all dogs are now registered companions, with little or no free-roaming populations [3,56,57]. The number of pet dogs continues to rise—from about 68 million in the early 2000s to over 78 million by 2012 in the U.S. alone—and similar growth is seen across the European Union, even as average household size declines [54,57,62]. This shift marks not only a demographic expansion but a cultural realignment. Dogs have moved from the margins of human life to its emotional centre. The Western world has never housed more dogs—yet this growth primarily reflects an urban, individualised model of companionship.

The transition from free-ranging to companion lifestyles involves clear trade-offs. While urban companion dogs are typically protected from environmental hazards and benefit from reliable access to nutrition and veterinary care—factors associated with improved physical health and longevity—these advantages coexist with constraints on social autonomy and peer continuity [63]. In this review, free-ranging populations are used as an ethological reference for species-typical social opportunities rather than as a “better welfare” comparator; we do not treat the two groups as directly equivalent across ecological conditions. Human–dog bonds provide meaningful interspecies support, yet they differ qualitatively from conspecific social regulation [64,65]. The present review therefore does not claim that free-ranging life represents a superior welfare condition overall, but rather focuses on how shifts in social structure may shape emotional regulation under otherwise improved physical conditions [66]. Accordingly, we frame these contrasts as hypothesis-generating and mechanism-oriented, and we explicitly note confounding differences (e.g., reproductive status, resource insecurity, disease load) as limitations of inference.

In industrialized urban settings, the dominant living arrangement for companion dogs is the single-dog household, with large-scale demographic studies consistently showing that the majority of pet dogs live without canine housemates [3,4,59,60,62].

### 4.2. Urbanisation as a Social Shift

Urban living has reshaped the ecology of companion dogs—imposing spatial, sensory, and legal limits far from the conditions in which canine social life evolved. For many urban dogs, daily life unfolds inside—apartments, small houses, and short walks replacing the open spaces their species once knew. They are kept on leashes, constrained by housing rules, and encounter few chances for spontaneous contact with other dogs [5]. Physical restriction is only part of the picture. Urban lifestyles—long working hours, compact living spaces, and the shortage of safe off-leash areas—further reduce opportunities for dog–dog interaction [5,18,67,68]. When dogs meet in dog parks, interaction bouts are often brief and dogs spend substantial time non-interacting [69,70]. This does not speak directly to the regulatory value of conspecific relationships, because high-turnover settings largely capture transient encounters among unfamiliar partners rather than repeated, low-conflict contact with familiar dogs, where affiliation and co-regulation can accumulate over time [71]. In such environments, short greeting bouts may reflect rapid assessment and distance regulation (often olfactory) rather than reduced social motivation [72].

### 4.3. Synthesis: Demography as Destiny

Across regions and cultures, one pattern stands out: in developed societies, most urban dogs now live as solitary companions [3,4,13]. This is not just a lifestyle change but a structural shift in the species’ social ecology [5,17]. For a group-living animal, solitary urban life means chronic underexposure—shaping emotion, stress regulation, and welfare [13]. Globally, this condition involves tens of millions of dogs—and an equal number of households that rely on them for support and routine [3,57]. Dogs who live alone and meet others only briefly experience a subtle, cumulative form of social deprivation [4,17]. Their emotional balance depends almost entirely on human mediation, with little access to the peer buffering found in natural canine societies [11,17]. Recognising this demographic reality is crucial for interpreting behaviour and welfare. The single-dog urban household is not just a cultural habit but a new social condition—one that tests the very systems dogs evolved to sustain emotional stability [17,73].

## 5. Social Life of Free-Ranging Dogs—The Forgotten Model of Canine Society

Free-ranging dogs live close to people yet remain largely independent. In loose groups they balance autonomy with cooperation—a flexibility that reveals what most urban dogs no longer experience: open, self-regulated social worlds.

### 5.1. Structure and Composition of Free-Ranging Dog Groups

Field studies from diverse free-ranging contexts (e.g., village dogs in Bali; urban street dogs in Morocco) describe autonomous social networks that retain structured, repeated associations despite substantial human presence [74,75]. Groups usually include two to ten dogs, fluctuating with mating or feeding seasons [11,12]. Membership is fluid: dogs come and go as food availability, safety, or reproduction change [61,76]. These groups resemble dispersed fission–fusion systems: membership shifts as resources and social conditions change, and individuals regularly join or leave [12,61]. This flexibility can preserve stable networks despite ecological variability—unlike the more rigid, human-imposed social boundaries typical of many urban companion dogs.

Despite this fluidity, groups maintain steady ties and recognisable roles—dogs rest, play, groom, forage, and sometimes help raise pups not their own [11,12]. Such bonds ease tension and prevent conflict. Hierarchies shift with context—dominance in feeding may not apply to mating or movement. Most groups show a soft, age-graded order, with older dogs usually taking precedence [11]. These fluid hierarchies reflect a system made for coexistence in changing conditions rather than control by force.

Reproductive status differs between the populations: free-ranging dogs are typically intact, whereas most companion dogs are neutered—a difference that can shape social behaviour and should be treated as a confound rather than a simple proxy for “reduced social motivation”. However, reduced sexually motivated behaviour does not imply reduced need for stable, affiliative dog–dog relationships. If anything, large-scale findings linking gonadectomy with higher social fearfulness or defensive responding in some populations [6,7,77] suggest that predictable, low-conflict social exposure may be especially important for emotional regulation. Reports of increased social withdrawal/reactivity in neutered females [78] and higher defensive aggression in neutered males in some datasets [77,79] support treating reproductive status as a confounder—rather than as a reason to restrict conspecific contact.

### 5.2. Cooperation, Affiliation, and Conflict Management

Free-ranging dogs show repeated affiliative contact and context-dependent cooperation, including documented alloparental care in some populations [80,81]. Variation in dominance stability can be associated with shifts in aggression patterns in free-ranging social networks [82]. Group presence can also alter responses to social challenges: in studies of urban free-ranging dogs, individuals in groups were more likely to approach and respond to human social cues (“bolder together”) than solitary dogs [83]. Within established groups, dogs usually manage tension through signalling and ritualised conflict behaviour rather than escalation [84]. Post-conflict affiliation and reconciliation have been described in dogs, although patterns vary across contexts [85]. By contrast, intergroup encounters tend to involve more agonistic displays and distance-regulating behaviour [80].

### 5.3. Comparative Implications for Companion Dogs

As a comparative model, free-ranging dogs illustrate a social ecology characterised by repeated exposure to familiar partners, flexible approach/avoidance, and group-level opportunities for affiliation and repair [11,12]. This comparator is not presented as a welfare ideal, but it helps specify which components of intraspecific social access and network continuity may be reduced or reshaped in urban companion dogs [1,86].

Free-ranging dogs keep social order mostly through ritualised signals, not force. Visual signalling studies show that small cues—posture, gaze, the rhythm of movement—are often enough to prevent conflict and maintain coordination during feeding or travel [84]. Vocal signals play a supporting role, serving more as distance markers or alerts than as threats. Placed against modern pet life, the contrast is often not “dogs meet few dogs” but “dogs meet many dogs with limited continuity.” In that sense, free-ranging networks function as a living reference ecology [87]: not a prescription, but a baseline for identifying what stable social access and low-conflict repetition can look like for a social species [84]. The stabilising components visible in free-ranging systems—repeated low-conflict contact, affiliative repair, and opportunities for social buffering—may be shortened or absent in typical urban routines.

## 6. The Social Reality of Urban Pet Dogs

Cities have become the primary habitat for companion dogs. Life here unfolds under constant human oversight—in apartments, elevators, narrow pavements, and fenced parks instead of open ground. They are stimulating places—full of sound and motion—yet autonomy is scarce, and most contact ends as soon as it begins. Urban dogs now live within a social world shaped by human routines and space: their days and contacts follow human rhythms more than canine ones.

Free-ranging dogs show a different logic. They stay organised and emotionally steady not through rules but through the feedback that flows naturally within the group. Relationships form by choice and context, regulating one another as they go. In such systems, social life is not decoration—it is what keeps them balanced.

Urban pets, in contrast, experience a shift from shared regulation to fragmented contact—a change that rewrites what being social means for them. What remains appears dense on the surface—dogs surrounded by people, meeting others every day—yet thin beneath it: contact without continuity, regulation without reciprocity.

This “thinness” in peer continuity has measurable behavioural and physiological correlates. Importantly, this does not imply that human support is negligible; rather, it suggests that human and conspecific interactions overlap only partly in social–emotional regulation. Neuroimaging and behavioural work indicates that dogs may rely on partly different regulatory strategies depending on whether the social partner is a human or another dog [64,65]. In some paradigms, dogs show more diverse facial signalling and more displacement behaviours in the presence of conspecifics [64], consistent with a species-specific socio-emotional “grammar” [88].

In parallel, the idea that dogs have fully adapted to socially restricted lives is difficult to reconcile with evidence from dogs living with limited conspecific contact. Even when basic care is adequate, restricted peer access has been associated with higher levels of cortisol metabolites and more displacement behaviours in some settings [66]. In intervention settings, studies that increase dogs’ opportunities for conspecific contact report subsequent reductions in stress-related measures and improvements in social outcomes [89]. At the population level, inadequate socialisation and low social exposure are associated with higher odds of social fearfulness [6]. This is consistent with a regulatory role for conspecific contact, but separating it from correlated factors (e.g., management style, environment, prior learning history) remains difficult.

### 6.1. Overview

Naturalistic observations suggest that city dogs meet often, but the meetings themselves are short and predictable. At off-leash parks, for instance, dogs spend around half their time alone and about 40% close to others, yet most interactions fade within minutes [72]. Greetings dominate interactions—quick bouts of mutual sniffing, usually lasting six seconds or so, then both move on [70]. The subtle postural shifts in these exchanges—tails settling, bodies relaxing—suggest their purpose is not excitement but reassurance, a way to prevent tension rather than invite it [70].

Controlled studies with unfamiliar pairs tell much the same story. Encounters are brief and shaped by who the dogs are—differ with sex, age, and neuter status. Females tend to start the approach, males linger in following and marking, while older dogs usually keep to themselves [90]. Aggression appears mainly in cramped or poorly designed areas, with space limitations and crowding as key triggers [91]. Urban sociability is dense in contact but thin in continuity—a world of encounters rather than relationships.

### 6.2. Patterns of Social Interaction and Affiliative Bonds

Affiliative bonds among dogs serve functions that reach far beyond companionship. They do more than create connection; they help dogs steady emotion, recover after conflict, and learn through one another’s feedback. In multi-dog households dogs tend to form stable relationships that resemble human friendships in their continuity and reciprocity [71]. Studies describing stable social hierarchies and affiliative structures [71,92] focus on dogs living in long-term groups, where continuity itself sustains stability. These structures are not strictly hierarchical. Recent findings by Vékony and colleagues further suggest that dominance in cohabiting dogs is a multifaceted construct, manifesting differently in competitive versus non-competitive scenarios, which emphasises that social roles are not static but context-dependent [93].

In practice, three broad patterns tend to recur: formal pairs that mix dominance with affiliation, egalitarian ones based on mutual friendliness, and a third, less defined group that does not fit either pattern. Each accounts for about a quarter of observed dyads. Observations from these studies show that dogs build lasting relationships. Such relationships are marked by steady proximity and coordinated behaviour, with little overt aggression—signals of familiarity and trust more than of rank. Yet the dogs in these studies lived in stable groups, a situation uncommon in the fragmented lives of most urban pets.

Play is central to these affiliative bonds. It supports coordination and recovery after tension, keeping partners in emotional sync. When familiar dogs play, bouts last longer and include pauses, self-handicapping, and shifts in position—gestures of adjustment rather than dominance [14,71]. Through play, dogs learn to balance arousal and restore connection [92]. It is not a by-product of friendship but one of its mechanisms. Brief encounters between unfamiliar dogs—the norm in cities—rarely develop into sustained play or attunement. This selectivity marks the social cost of urban living: dogs surrounded by many conspecifics yet forming few meaningful ties. Even in structured environments, familiarity still shapes emotional balance. Dogs that live with steady partners recover from tension more quickly and show fewer arousal spikes during play [14]. Social competence depends not on quantity but on continuity—the element missing from most urban lives. Many city dogs live in socially crowded yet relationally sparse worlds: they meet many dogs but know almost none. The result builds slowly—less social confidence, more watchfulness, greater dependence on human cues for stability.

### 6.3. Developmental Roots of Social Dysregulation

Social regulation in dogs is not a fixed instinct. It grows through practice—through many small exchanges with steady, socially skilled adults. In natural groups, older dogs moderate the young: they ease arousal, redirect play, and show how to repair tension when it appears [94]. This kind of tutoring, an alloparental form of emotional coaching, teaches puppies to read signals, adjust to feedback, and find calm again after conflict [11,95,96]. Across such encounters, they absorb the social rhythm of their species—a way of staying attentive and measured instead of impulsive. In cities, that system has almost vanished. Most puppies grow up surrounded by people but see few consistent canine examples. Juveniles meet peers, sometimes stressed or undersocialised adults, but rarely mentors [97,98]. Without that steady modelling of restraint and repair, social feedback turns patchy—uneven exposure, mixed messages, little correction [11,12,14,99]. By adolescence, many dogs show quick arousal and slow recovery—they need constant cues, constant help [6,7,73].

This loss builds quietly. Each missed moment of canine feedback—a cut-off play, a misread signal, a familiar partner gone—can slow the development of coping skills and make flexible social adjustment harder [22,100]. Gradually, strategies harden: avoidance takes the place of negotiation, watchfulness replaces curiosity, attachment overshadows autonomy [101,102]. What from the outside looks like obedience or calm can just as well be emotional contraction—a learned inhibition born of missing, species-typical regulation [103,104].

The same developmental rule shows up across social mammals. Among primates, ungulates, and other social mammals, early contact with steady companions builds resilience and flexibility later in life [82,104]. Dogs probably share that pattern, although modern rearing often breaks it. Breeding and adoption routines—early separation, limited peer contact, and fragmented daily life—remove the scaffolding that once held canine societies together. The outcome is contradictory—dogs bred for sociability, yet deprived of the conditions that allow it. They keep the drive to cooperate but lose the practice, and that mismatch runs deep into emotional development.

Dogs may be bred for human sociability today, but the groundwork for their emotional development starts in early contact with other dogs [22,105]. In this sense, pro-social tendencies and social needs are not identical: cooperation with humans was shaped by selection [106], but the architecture of co-regulation remains rooted in canine social life [39,100].

### 6.4. Emotional and Behavioural Consequences

Most city dogs live with frequent exposure to unfamiliar dogs—they see many conspecifics but rarely build familiarity. Encounters are usually short and often polite, but they do not always turn into repeated contact with the same partners [69]. That gap matters. Social learning and regulation depend on repetition and predictability—on knowing what to expect [6,7]. When interactions are mostly one-off and uncertain, many dogs adopt low-risk strategies: they freeze, stay quiet, and monitor. What looks like calm compliance can reflect withdrawal—a way to stay stable when social contact is unpredictable [22]. Limited access to familiar partners may therefore contribute to faster arousal escalation and slower recovery [22,39,100].

Studies link this pattern with anxiety, and a growing dependence on human cues [6,7]. Dogs deprived of regular affiliative feedback lose opportunities for co-regulation [39]; their arousal runs higher during social exposure and recovery slows after stress [22,100]. Physiological findings point the same way: higher cortisol [39], displacement behaviour, less exploration, and a kind of low-level watchfulness that never fully switches off [6,7,64]. Even positive encounters, such as play in dog parks, carry their own constraints. They unfold in crowded, noisy settings where arousal runs high and human intervention often cuts interactions short [91,107]. These experiences offer contact but little continuity, reinforcing a pattern of social density without depth.

### 6.5. Synthesis: The Managed and Ecologically Constrained Social World

When the evidence is viewed together, a clear picture emerges: city dogs live within social systems shaped as much by human design as by their own species’ needs. Most depend on people to reach other dogs at all. Urban dogs live within compressed social spaces—shaped by architecture, regulation, and by how much human tolerance allows. It is a simple but striking tension: surrounded by others, yet short of the autonomy and continuity that make social life self-regulating.

## 7. The Fragmented Social World of the Urban Dog—A Theoretical Synthesis

### 7.1. From Social Flexibility to Social Deficit

An unusual social flexibility—a capacity to read, adjust, and find rhythm across species boundaries—was acquired by (or demanded from) dogs through the process of domestication. It let them regulate behaviour within both canine and human groups [1,14]. Cooperation depended on feedback rather than command—a pattern sustained by familiarity and trust [11]. In modern cities, that balance has fractured. Dogs still carry the neural and behavioural equipment for collective regulation, but the social scaffolding that once supported it has thinned. Most emotional feedback now flows through a single relationship—with the human guardian—whose tone mirrors the tension and instability of human social life [6,7]. Dogs remain profoundly social, yet now depend on humans for safety and regulation alike.

### 7.2. Theoretical Integration: Attachment, Stress Regulation, and the Ecology of Safety

The breakdown of shared regulation reveals how deeply emotion depends on relationship. As networks thin out, the sense of safety which is usually built on mutual cues begins to fade. What follows is not weakness in the individual but a weakening of the attachment structures that keep social species in balance.

Emotional regulation in social species does not arise within individuals but within systems of attachment. Secure relationships provide predictability—a sense that signals elicit predictable responses from others, and that stress does not have to be faced alone. Across mammals, this pattern underpins resilience: when familiar partners respond consistently, the nervous system learns safety instead of vigilance [16,82].

For dogs, this architecture has always been layered. Attachment is distributed rather than singular, extending across both conspecific and human partners. Regulation occurs through overlapping exchanges—play, touch, shared rest—each reinforcing emotional stability [1].

### 7.3. Implications for Welfare, Behavioural Therapy, and Research

If emotional regulation depends on social architecture, then behavioural problems cannot be understood—or treated—apart from it. Many problems that appear as behavioural disorders—reactivity, fear, separation distress, or over-attachment—may instead reflect social deprivation. They are ways of coping in environments that limit autonomy and disrupt shared regulation. Seeing these patterns as personal failings misses the broader conditions that sustain them. From this angle, therapy is less a matter of fixing behaviour and more of rebuilding shared regulation.

The emphasis moves away from obedience toward steadiness—from control toward a capacity for mutual adjustment.

Interventions such as structured peer contact, predictable walking routines, or supervised time with familiar dogs can function as therapeutic supports rather than ‘enrichment’ in the narrow, object-based sense. Environmental enrichment can improve welfare in kennelled dogs, but social contact and structured routines target regulation more directly than toys or novelty alone [108,109]. In applied settings, low-intensity social support can be framed as a welfare intervention aimed at reducing stress and supporting adaptive coping, rather than as optional stimulation [110].

For welfare science, the task may be to widen its lens beyond physical conditions and activity levels. Available evidence suggests that social predictability and repeated, low-conflict contact can support emotional regulation, but the field still lacks direct tests in typical urban single-dog households. From this perspective, welfare may partly depend on continuity—familiar partners, stable routines, and repeated encounters that allow dogs to anticipate social outcomes. We therefore propose that small, predictable “micro-communities” of trusted relationships could function as a practical welfare scaffold, potentially buffering arousal and supporting recovery in ways that space or stimulation alone may not achieve.

In urban settings, welfare is influenced not only by physical conditions but also by how predictable daily routines are and how stable social exposure is over time. This suggests that improving welfare may require changes at the level of environments and practices—not only training.

## 8. Methodological Challenges and Research Gaps

The fragmented social world of the urban dog may be not only an ecological or psychological condition but may also reflect a methodological one. For decades, behavioural science has often favoured models that isolate rather than connect—brief encounters, discrete events, dyads detached from the networks that give them meaning [86]. As a result, much of what we know about dogs comes from contexts shaped by human direction: in training sessions, attachment tests, or shelter routines [10]. Spontaneous architecture of dog society with its informal rules, alliances, and social rhythms—remains comparatively unmapped [86].

Current methods often code short behavioural bouts, which can capture detail but limits inference about longer-term continuity of affiliation and co-regulation; repeated naturalistic observation in everyday settings (e.g., sidewalks and parks) is therefore likely necessary [86].

### 8.1. Underrepresentation and Sampling Bias in Companion-Dog Research

Most empirical knowledge about canine social behaviour comes from contexts that are convenient, and may not be representative. Shelters, day-care facilities, training centres, dog parks, and multi-dog households dominate the literature because they make observation easy [69,72,91,104].

Yet these settings represent only a fraction of the companion-dog population and can differ from the social realities of single-dog urban households, creating structural sampling bias [69,72,91]. Dog parks capture high-density, brief encounters [69,70,72] while shelter studies reflect confinement-related stress rather than ordinary routines (Table 2) [66,104].

Baseline mapping remains limited: how many dogs a typical city dog meets, which contacts become repeated relationships, and how stable those ties are over time [87]. Without such mapping, claims about development, stress, or deprivation can rest on assumptions rather than measured social exposure.

Olfactory communication is also comparatively underrepresented in companion-dog work. Free-ranging studies indicate that scent marking and scent discrimination structure territoriality, familiarity, and social tolerance [111], while companion-dog research often prioritises visual/tactile interaction [112,113]. Because olfactory cues do not require co-presence, they may support recognition and low-intensity social buffering that standard observation misses; incorporating marking, overmarking, and scent-based investigation may therefore improve network mapping [111].

Finally, many experimentally informative manipulations (e.g., restricting contact or altering access) are ethically difficult to justify in companion dogs, which increases reliance on naturalistic observation and guardian reports and limits causal tests in everyday urban ecologies.

### 8.2. Existing Methods for Assessing Dog–Dog Relationship Quality

Researchers studying canine relationships have already developed several ways to assess how these bonds form and persist over time. Most of this work comes from semi-controlled environments—multi-dog households, day-care groups—where researchers could follow repeated interactions over time. They used detailed ethograms to record the frequency, direction, and symmetry of affiliative and agonistic behaviours: approach and retreat, proximity, greetings, play, avoidance, reconciliation [71,92,114].

These datasets support relationship classifications (e.g., formal vs. egalitarian) and highlight play as a sensitive indicator of relationship quality [14,92]. Dominance-focused measures add information about rank stability and submissive signalling [93,114].

Even when social-network analysis is applied to dogs, its roots lie in ecology and epidemiology—tools designed to trace the spread of pathogens or information, rather than to capture relationship quality or affective regulation [115]. Networks based on co-occurrence can miss functional distinctions—contact is not affiliation and proximity is not trust—so recent work has begun to combine structure with indicators of relationship quality [11,92,93]. Because dogs interact extensively through olfaction, repeated investigation of specific scent traces and marking patterns may index familiarity or partner preference that co-presence metrics overlook [111].

Limited quantitative work offers a glimpse of what is missing: among urban free-ranging dogs, social networks averaged about twelve companions, including three to six core associates [12]. Pet dogs, by contrast, averaged only four companions and one to three close ties—a clear contraction of social structure in those samples [12]. But these data came from cultures with high public access and mobility, unlike the spatial constraints of Western cities. They reveal the scale of compression but also the limits of current frameworks. Yet no comparable mapping exists for companion dogs. Their social networks remain poorly described—who they know, how often they meet, and what those encounters mean for emotional stability are still uncharted [86].

**Figure 1:**
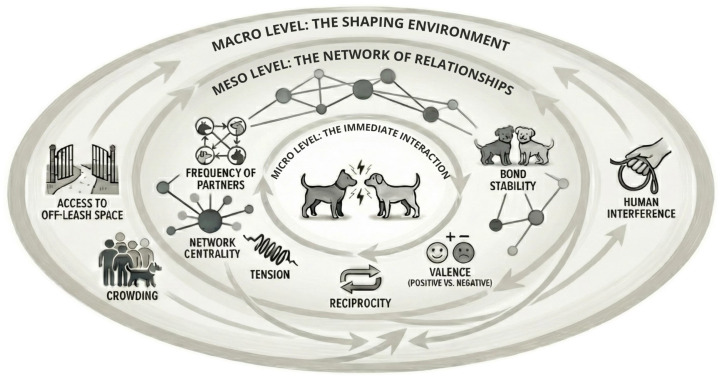
Micro: short-term shifts during encounters; meso: repeated meetings and relationship stability; macro: spatial constraints and human management shaping contact opportunities. Arrows indicate dynamic feedback loops and mutual influences between components at each level.

Accordingly, a socially grounded approach needs two steps: (i) describe repeated exposure (who meets whom, how often, and where), and (ii) characterise relationships (which partners recur, how stable ties are, and what their affiliative tone and reciprocity look like). Figure 1 adopts Hinde’s separation of interactions, relationships, and social structure to organise these measures [115].

Multi-dog households are not treated as a solution: available evidence is mixed, and any benefits likely depend on the quality and stability of within-household dog–dog relationships rather than dog number per se [116,117,118,119,120].

### 8.3. Comparative Models of Social Co-Regulation in Mammals

Across other social mammals, social and emotional co-regulation has been examined through behavioural and physiological measures. In primates, close bonds buffer stress, with oxytocin down-regulating HPA activity and reducing arousal [121]. Elephants show tactile reassurance and post-distress affiliation, indicating group-level coordination of calm [32]. In rodents, grooming and close contact are linked to oxytocin release and lower stress and anxiety [33], consistent with a conserved mechanism of social buffering via proximity, touch, and coordinated behaviour [32,33,121,122].

In primates and rodents, shifts in oxytocin and cortisol trace co-regulation—stress softening through contact [33,34,51]. Among primates, reconciliation after conflict is often treated as a proxy for emotional repair, though it does not always map cleanly onto it [31]. In elephants or horses, coordination and touch signal low-intensity social maintenance—a return to balance within the group [32]. Rodent work adds the idea of emotional contagion through scent or gentle pressure [123]. Across species, the pattern is recognisable enough: steadiness grows from shared regulation more than from control [124].

Modern network science combines structural and affective indicators—frequency, duration, perceived closeness, trust, and access to support [125,126,127]. Although based on self-report, the same logic applies to nonverbal species. For dogs, relational quality could be operationalised through stability of preferred partners, symmetry in play, tolerance in confined spaces, and speed of recovery after conflict—functional analogues of trust and reciprocity. Integrating such measures with network metrics would move canine social research from describing contact to mapping relationship.

Behavioural observation combined with network tools restores dogs to the broader study of social systems—one in which affiliation is not a by-product of proximity but an adaptive structure of regulation and trust.

Table 3 outlines the core functional dimensions that describe the quality of social relationships in dogs.

Human work on social buffering and co-regulation often operationalizes these processes via attachment constructs and physiological indices; comparable, validated measures are still limited in dogs, and this review therefore focuses on dog–dog relationships.

### 8.4. Toward a Multi-Context Ethology of Urban Dogs

To understand the social world of urban dogs, research may need to follow behaviour across the main everyday contexts in which dogs move (home, walks, parks), rather than sampling a single setting (e.g., only dog parks or day-care) [60,98].

Olfactory information can add another layer to this picture. Patterns of marking, counter-marking, or repeated interest in the same scent traces may point to familiar partners or ongoing relationships that never appear in direct encounters [111].

Wearable sensors and lightweight physiological monitors can record stress patterns without disrupting natural behaviour [37,38]. Video-based microanalysis makes it possible to code fine details of affiliative behaviour—gestures, moments of synchrony, or brief repair sequences. Complementary data can also be gathered through guardian-reported measures, which, although less precise, make it possible to capture large-scale patterns of social contact and emotional stability across diverse urban contexts. Together, these tools can show how social stability or fragmentation affects well-being.

To make this idea measurable, it is worth defining spatial zones roughly matching the sensory and behavioural reach of canine communication—a working frame rather than a fixed rule (Table 4).

### 8.5. Practical Implications: Rebuilding Micro-Communities

Putting social restoration into practice may require changes in everyday handling and expectations, not only urban design [98,107]. Most city dogs live surrounded by other dogs but have limited control over when and how contact happens; this can turn social exposure into something to manage rather than a resource for regulation. In practice, a shift toward allowing low-conflict, predictable social micro-interactions (sniffing, brief greetings, distance adjustment) may help make encounters calmer and more predictable over time [97,98].

Micro-communities are unlikely to emerge without deliberate facilitation: repeated, low-conflict exposure on walks (stable routes, predictable encounters, sufficient distance) can support familiarity and reduce escalation [97,98]. Education is therefore a practical welfare lever—guidance for guardians and the public can shift norms from interrupting contact toward managing space and time so that dog–dog interaction remains voluntary and safe [63].

### 8.6. Ethical and Welfare Reflections

Observing companion dogs in everyday urban spaces requires minimising interference and ensuring voluntary participation. Where physiology is measured, non-invasive methods (e.g., heart rate variability collars; cortisol from saliva or hair) should be used to avoid constraining movement or interrupting social contact [37,38,40]. Combining brief observation with guardian reports can reduce intrusion while retaining contextual information.

## 9. Conclusions

Dogs regulate in relationships: familiar partners and repeated, low-conflict contact can support recovery and reduce vigilance. In urban environments, social thinning may shift regulation toward the human–dog dyad and increase reliance on human management.

For welfare science, this implies that sociability is not an optional add-on but a core component of the relational infrastructure supporting emotional stability. Research that maps how dog–dog ties form, recur, and persist—and how routine and spatial design shape access to them—can clarify when contact functions as regulation rather than mere co-presence.

Urban dogs may mirror a wider pattern of urban social life: high co-presence with limited continuity.

Future studies should follow dogs across everyday contexts over time, linking encounter structure, relationship stability, and environmental constraints. This may help bridge behavioural research with welfare practice and urban planning by identifying conditions that protect dogs’ capacity for social life.

## Figures and Tables

**Table 1:** Global patterns of dog social ecology.

Region/Country	Dominant Dog Population Type	% Free-Ranging	% Single-Dog Households	Key Urban Constraints
India/Brazil/Africa [12,53]	Community and semi-owned dogs integrated into human environments	70–90%	N/A	High population density, open access to streets, shared resources
Western Europe/US [3,54,55]	Fully owned, confined companion dogs	<5%	70–80%	Strict leash laws, limited off-leash space, apartment living
Global Trend [56,57]	Shift from free-ranging to owned populations	–	Increasing single-dog ownership	Urban density, legal control, risk-averse culture

**Table 2:** Methodological scope and gaps in companion-dog research.

Existing Method	Typical Scope	Populations	What It Captures	What It Misses
Behavioural ethograms	Long-term, narrow scope	Multi-dog households, day-cares, shelters	Dominance and affiliation patterns	Broader social context, network structure
Owner or guardian surveys	Short term, broad scope	Urban pet dogs	Focused on human–dog relationship. Dyadic interaction, perceived behavioural issues	Broader social context, network structure
Dog-park observations	Short term, broad scope	Urban pet dogs	High-density play and conflict behaviour	Stable relationships, network structure
Network analysis	Long term, broad scope	Free ranging dogs	Structure and frequency of contact	Emotional valence, reciprocity, stability

**Table 3:** Functional dimensions of social relationship quality in dogs.

Functional Dimension	Theoretical and Interpretive Meaning	Example Behavioural/Relational Indicators	Possible Data Sources or Methods
Tension (arousal/constraint)	Expresses the degree of physiological and behavioural arousal within relational patterns; high tension indicates low perceived safety or regulatory overload	Body stiffness, scanning, speed of movement, micro-pauses (“freezes”), displacement behaviours	Observation, video coding, heart rate variability or cortisol
Relational valence	Reflects the overall affective tone and balance of participation within interactions; indicates whether exchanges are cooperative, neutral, or conflictual	Frequency of positive vs. negative behaviours, reciprocity, ease of repair after conflict	Field observation, video coding, guardian reports
Bond stability	Describes the range and heterogeneity of dogs encountered within the individual’s social environment; includes both brief and recurrent contacts	Number of distinct partners encountered over a defined period, variety of contexts (home, park, walks), proportion of novel vs. familiar dogs	Structured observation, short guardian logs of daily encounters, annotated video samples
Social integration	Represents the individual’s embeddedness within a broader social network; linked to access to social support and group cohesion	Number and diversity of partners, network centrality, isolation, bridging roles	Social network analysis, observational mapping

**Table 4:** Spatial zones of canine social contact.

Contact Zone	Description	Typical Behaviours or Signals
Distal	Interaction initiated at a distance; communication occurs through posture, orientation, and gaze (usually beyond 5–10 m, depending on terrain and visibility)	Sustained gaze, curved approach, intentional stop, body angling, tension release through movement, scent marking
Mid-range	Mutual awareness and approach within visible and olfactory range, without physical contact (typically 1–5 m)	Parallel orientation, slow approach, posture signalling intent, relaxed stance, micro-pauses, scent marking and overmarking
Proximal	Close proximity allowing brief or partial physical contact (within roughly 1 m or one body length)	Sniffing, nudging, shoulder or side contact, short affiliative pauses, shared resting area
Tactile	Full physical contact; overlap of personal space or sustained touch (direct contact, 0 m)	Play, grooming, leaning, lying together, affiliative body pressure

## Data Availability

No new data were created or analysed in this study. Data sharing is not applicable to this article.
